# Study of a Nanostructured Co-Doped SnO_2_ Sensor for Hydrogen Peroxide Vapor Detection Using Impedance Spectroscopy

**DOI:** 10.1021/acsomega.5c00917

**Published:** 2025-04-06

**Authors:** Gohar Shahnazaryan, Mikayel Aleksanyan, Artak Sayunts, Zarine Simonyan, Rima Papovyan, Gevorg Shahkhatuni

**Affiliations:** Center of Semiconductor Devices and Nanotechnologies, Yerevan State University, 1 Alex Manoogian, 0025 Yerevan, Armenia

## Abstract

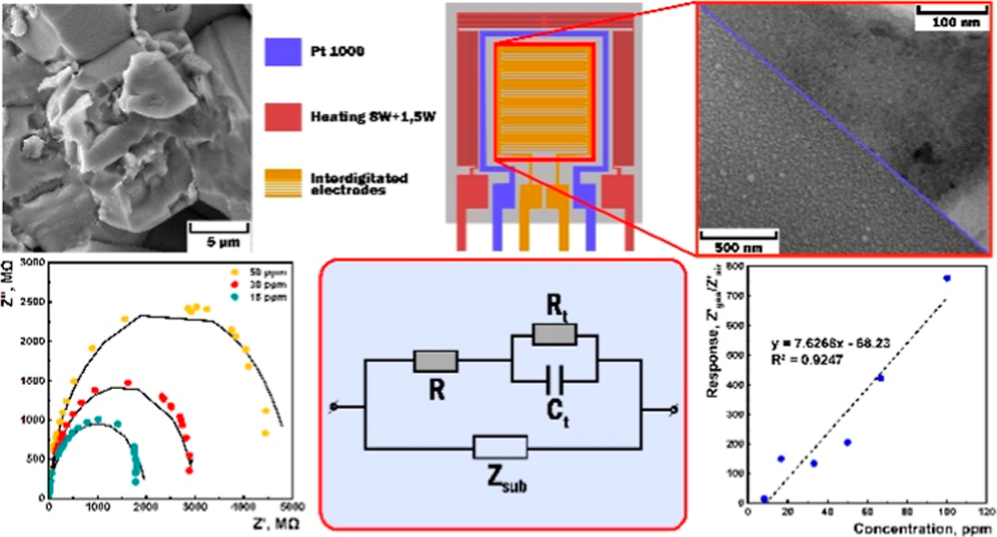

A hydrogen peroxide vapor (HPV) sensor based on SnO_2_ doped with 1.3 at. % Co thin film has been fabricated using the high-frequency magnetron sputtering method. The thickness of the SnO_2_ ⟨Co⟩ thin film was measured and the surface morphology was examined using the thickness measurement profilometer and scanning electron microscopy, respectively. The crystalline properties of the sensing material were revealed by transmission electron microscopy. The response, current–voltage, and impedance characteristics of the sensor were measured in the air and in the presence of various concentrations of HPV at 25–200 °C. An equivalent electrical circuit for the manufactured sensor structure was proposed, and the parameters of its constituent elements were determined. Furthermore, fitting frequency dependences of impedance were calculated. It was shown that charge transfer in the SnO_2_ ⟨Co⟩ thin film was regulated by the processes mainly occurring at the grain boundaries of the gas-sensing film.

## Introduction

The contemporary existence of humankind is inextricably linked to using gas sensors, the necessity of which is increasing annually. Their applications are widespread, enabling real-time monitoring, detection, and control of potentially harmful and explosive gases. Semiconductor metal oxide (SnO_2_, ZnO, Fe_2_O_3_, TiO_2_, CuO, WO_3_, etc.) sensors, the operation of which is based on the adsorption of target gas molecules on the active surface; occupy a distinctive position among the multitude of gas sensor types. These sensors are considered to be one of the most extensively researched due to several factors, including their high sensitivity and selectivity, simplicity, small size, and low cost. Despite the significant achievements made, the investigation of metal oxide sensors remains a current and active area of research, and efforts are ongoing to improve their gas-sensing parameters. The advent of nanotechnologies has opened new vistas in the field of metal oxide gas sensor development. The potential for utilizing nanoscale materials in sensors, which offer a high surface-to-volume ratio, has opened avenues for enhancing the sensitivity of sensors and surmounting the challenges associated with speed, stabilization, and optimization of other key parameters. Presently, there is a search for advanced gas-sensitive materials and the development of new technologies for their production. Concurrently, efforts are underway to enhance the parameters and characteristics of the existing sensors. These works are developing intensively today to reveal the gas-sensing mechanism of the material, to identify suitable dopants, to determine their optimal concentrations, and to refine the nanostructured metal oxides via optimization of the production techniques.^1−12^

Metal oxide materials and their composite nanostructures are widely used in the design of modern nanoscale devices, resulting in optical, gas, and other detectors.^13−19^ Among the metal oxide materials used in sensors, SnO_2_ is considered the most widely used and well-studied as it is highly sensitive to both reducing and oxidizing gases.^20,21^ It is a semiconductor with a fairly large band gap energy (3.6 eV) and has a rutile tetragonal crystal structure. Its physical parameters are quite favorable for use as a gas-sensitive element (melting point: 1630 °C, molar mass: 150.708 g·mol^–1^, density: 6.95 g/cm^3^ (20 °C), lattice constant: *a* = 4.737 Å, *c* = 3.185 Å (α = 90°, β = 90°, γ = 90°), heat capacity: 52.6 J/mol·K).^22,23^ Besides, Co_2_O_3_ is quite suitable as a dopant material, as it has a fairly high chemical activity and is capable of tanning the band gap energy of the main gas-sensitive material. Cobalt oxide is quite stable to environmental toxic substances and chemical gases and has an acceptable combination of physicochemical parameters (melting point: 895 °C, molar mass: 165.86 g·mol^–1^, density: 5.18 g/cm^3^ (20 °C), crystal structure: corundum, magnetic susceptibility: +4560.0 × 10^–6^ cm^3^/mol).^24^

Typically, pure metal oxides exhibit rather poor response to target gases due to their almost immeasurably high electrical resistance, low chemical activity, and difficult redox reactions. For this reason, various dopants are introduced into the basic metal oxide material, which vastly improves gas-sensing properties, such as sensitivity, speed, selectivity, and stability. Here, the main SnO_2_ gas-sensitive material in its pristine state exhibited high resistance and extremely low sensitivity and selectivity to hydrogen peroxide vapor (HPV).^25,26^ On the other hand, Co_2_O_3_ is known as a good dopant that dramatically improves the performance of sensors against oxidizing gases.^27^ The effective combination of these two materials has led to the high sensitivity, stability, and rapidity of the main SnO_2_ ⟨Co⟩ structure to the detection of HPV.

The aforementioned principles also apply to sensors specially designed for the detection of hydrogen peroxide (H_2_O_2_). These sensors are employed in various industrial and commercial contexts, including the food, textile, paper, and pharmaceutical industries, the production of cleaning agents and disinfectants, environmental monitoring, analytical chemistry, security sectors, and clinical diagnostics. In these fields, they are used to quickly and accurately diagnose a variety of diseases and monitor the effectiveness of a treatment. Given the potential dangers of hydrogen peroxide to human life when its concentration exceeds the maximum permissible level, the detection of H_2_O_2_ and the determination of its concentrations remain relevant. A variety of sensors have been developed for the detection of HPV, using a wide range of techniques including amperometry, colorimetry, luminescence, fluorescence, near-infrared spectroscopy, and electrochemical and chemoresistive methods.^28−38^

In order to enhance the sensor performance, it is essential to gain a comprehensive understanding of the underlying mechanisms governing charge transfer in metal oxides and their interaction with the target gas. In this context, studies using impedance spectroscopy to investigate the electrical properties of metal oxide semiconductor interfaces at different frequencies provide insight into the mechanisms governing their gas sensitivity. Impedance spectroscopy is not a novel method, although it has been applied since the 1940s for the analysis of electrochemical systems. In addition to the information that can be obtained from direct current (DC) measurements, sensor studies by alternating current (AC) can quantify the contributions to the conductivity of individual regions of the sensor structure at different operating temperatures and in different environments. Furthermore, they can evaluate the magnitude of certain parameters such as the height and width of energy barriers and investigate the influence of various factors on gas-sensing behavior, including grain size, temperature, and energy barriers. This allows for a deeper understanding of the gas-sensing mechanisms of the sensor performance.^39−43^

Impedance spectroscopy is not a commonly used method in the sensor field due to the inherent difficulties in analyzing the acquired frequency characteristics. To reveal the phenomena of interest, it is necessary to make impedance measurements over a wide range of frequencies and temperatures, at different displacements and amplitudes of the sinusoidal signal and in different surrounding atmospheres. For a comprehensive analysis of the processes occurring within a sensory structure, the obtained impedance data are represented in different coordinates (Nyquist, Bode curves, etc.). The main difficulty in applying impedance spectroscopy is that different electrical circuits may exhibit identical impedances at all frequencies. Thus, in practice, it is usually possible to find several equivalent circuit diagrams that numerically match a given data set, but only one of them can match the actual electrical behavior of the sensor. To select the appropriate equivalent circuit, it is necessary to take into account the physical and chemical characteristics of the system as well as any additional information about the properties of the sensing material obtained using alternative methods. It should be noted that advancement of technical and software supporting methods in recent decades has allowed significant reduction in the time required for the acquisition and processing of experimental data, which, in turn, increased the demand for impedance spectroscopy.^44−48^

This work aimed to fabricate an HPV sensor based on a Co-doped SnO_2_ nanostructured thin film and investigate its gas-sensing behavior by impedance spectroscopy combined with DC measurements. The novelty of the work is attributed to the fact that the HPV sensor, designed and manufactured using inexpensive, simple, and reproducible technological methods, was thoroughly investigated not only by the resistive method but also by the impedance spectroscopy using a high-frequency signal, as a result of which the most important factors affecting the gas-sensing parameters of the sensor were uniquely identified.

## Experimental Section

### Fabrication of the Sensor

**Figure 1:**
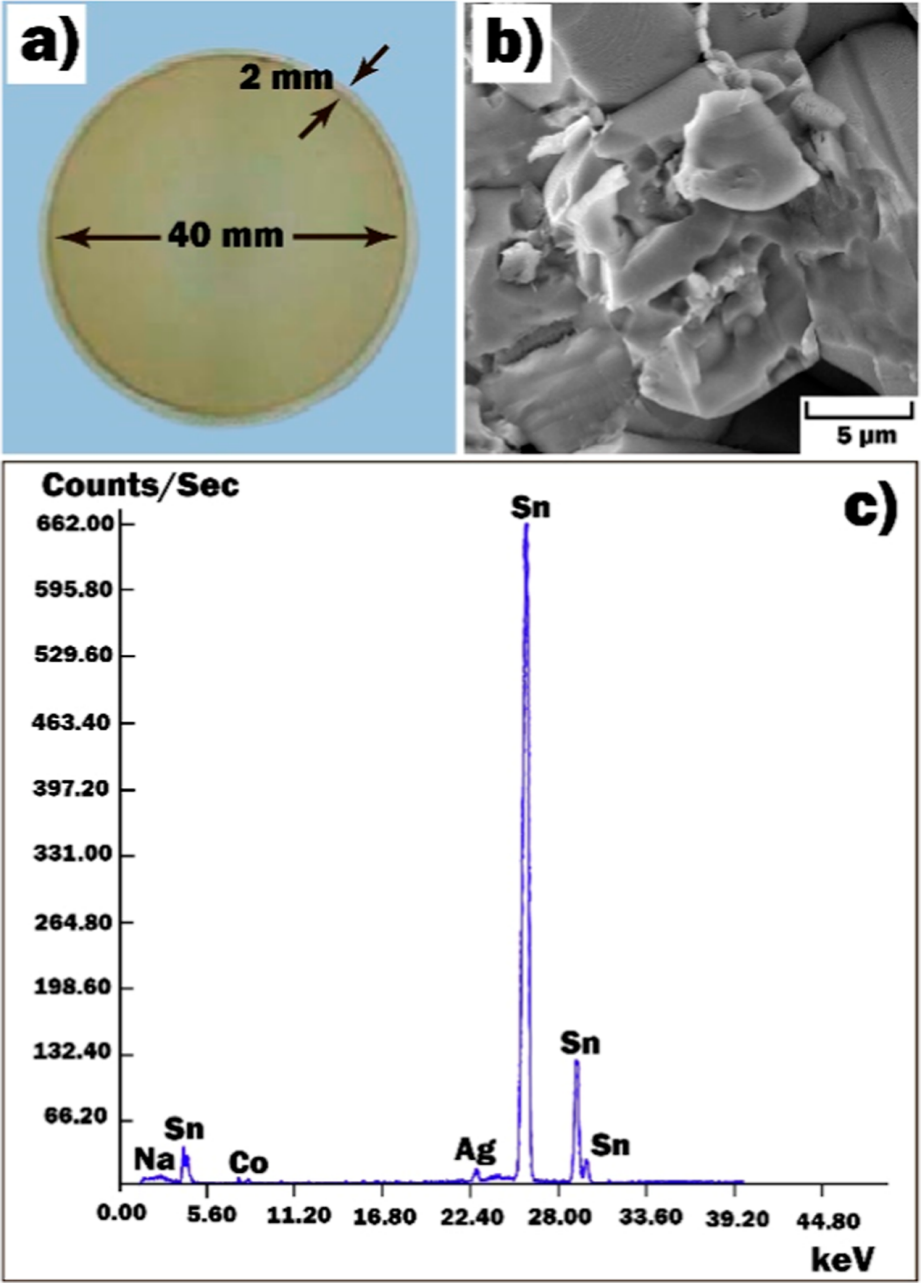
(a) Actual photo of the polycrystalline SnO_2_ ⟨Co⟩ target, (b) SEM image of the target, and (c) XRF analysis data of the target.

The SnO_2_ ⟨Co⟩ thin film sensitive to HPV was fabricated using the high-frequency magnetron sputtering method. This technique offers several advantages, including technological simplicity, high speed (1–10 nm/s), film uniformity, possibility of deposition of various types of films (from metallic to dielectric), excellent adhesion, and ability to control film properties. The polycrystalline target utilized in the magnetron sputtering process was synthesized earlier by using a high-temperature solid-state reaction (Figure 1a). Here, the appropriate amounts of high-purity SnO_2_ and Co_2_O_3_ powders (99.9%, Alfa Aesar) were used. The fabrication process of the ceramic target is thoroughly described in our previous works.^37,49,50^ It is important to note that the maximum synthesis temperature for the n-type SnO_2_ ⟨Co⟩ semiconductor target reached 1100 °C. Below this temperature, the solid-phase reaction partially occurred, which led to incomplete doping, and the target material did not have much mechanical strength and measurable electrical resistance. At higher temperatures (>1100 °C), the temperature of the metal oxide composite approached its melting point, where the loss of the crystalline properties became more likely. The microstructure of the target (Figure 1b) was examined by scanning electron microscopy (SEM) (MIRA3 TESCAN) spectrometry. The SEM image revealed the existence of intergrain spaces indicating the porosity of the target. The X-ray Fluorescence (XRF) analysis data (Niton XL3t GOLDD+ XRF Analyzer) confirmed that the actual amount of cobalt in the synthesized target was 1.3 at. % (Figure 1c).^37,49,50^ The XRF is a relatively inexpensive and fast-to-use technique that does not damage the crystal structure or surface of the examined sample. It is an accepted method for quantitative, qualitative, and semiquantitative analysis of nanomaterials.^51^

As a sensor substrate, factory-designed multisensor plates (TESLA BLATNA, Czech Republic) were used. For the deposition process, the following technological regimes were selected: 60 W high-frequency magnetron generator power, 20 min deposition duration, 200 °C substrate temperature, and 7 cm target-to-substrate distance. Catalytic palladium nanoparticles were deposited on the surface of the deposited SnO_2_ ⟨Co⟩ thin film using the DC magnetron sputtering method with a deposition time of 3 s. At the final fabrication stage to obtain homogeneous films, eliminate mechanical stresses, and stabilize the resistance, the sensor structure was annealed in the air at 350 °C for 4 h.

### Sensor Geometry

**Figure 2:**
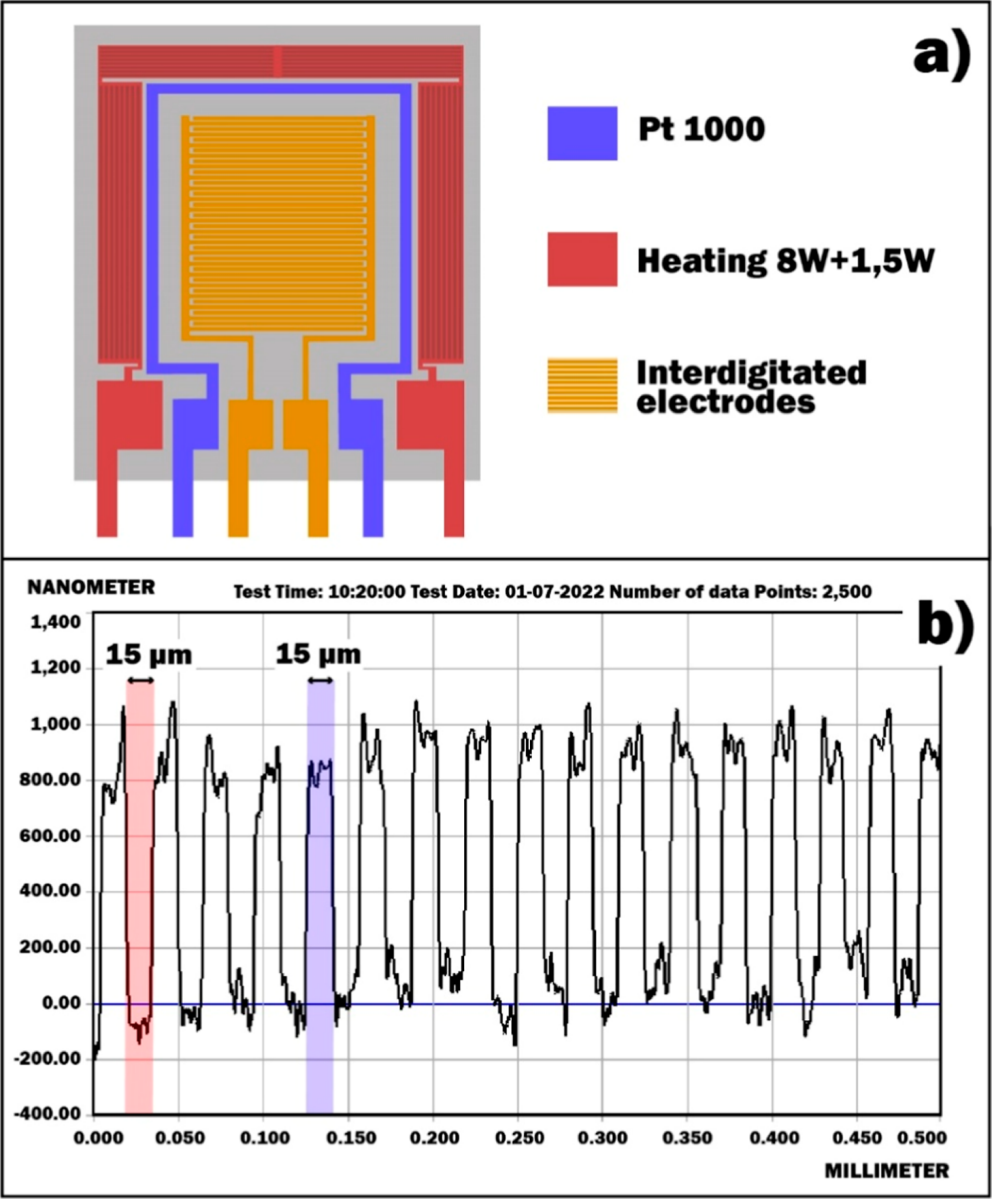
(a) Schematic representation of the multisensor platform and (b) profile of IDEs.

The schematic illustration of the multisensor platform is shown in Figure 2a. The platform integrated a temperature sensor (Pt 1000), a heater, and platinum interdigitated electrode structures (IDEs) printed on the alumina substrate. The heater and temperature sensor were coated with an insulating glass layer. The profile of the IDEs was revealed using the Alpha-Step D-100 profiler (KLA Tencor, Milpitas, CA, USA) (Figure 2b). The widths of the electrodes and the gap between them were both 15 μm. The contact thickness was in the range of 800–900 nm. A SnO_2_ ⟨Co⟩ nanostructured layer was sputtered onto the IDEs with an area of 4.76 mm^2^.

### Measurement Methodology

**Figure 3:**
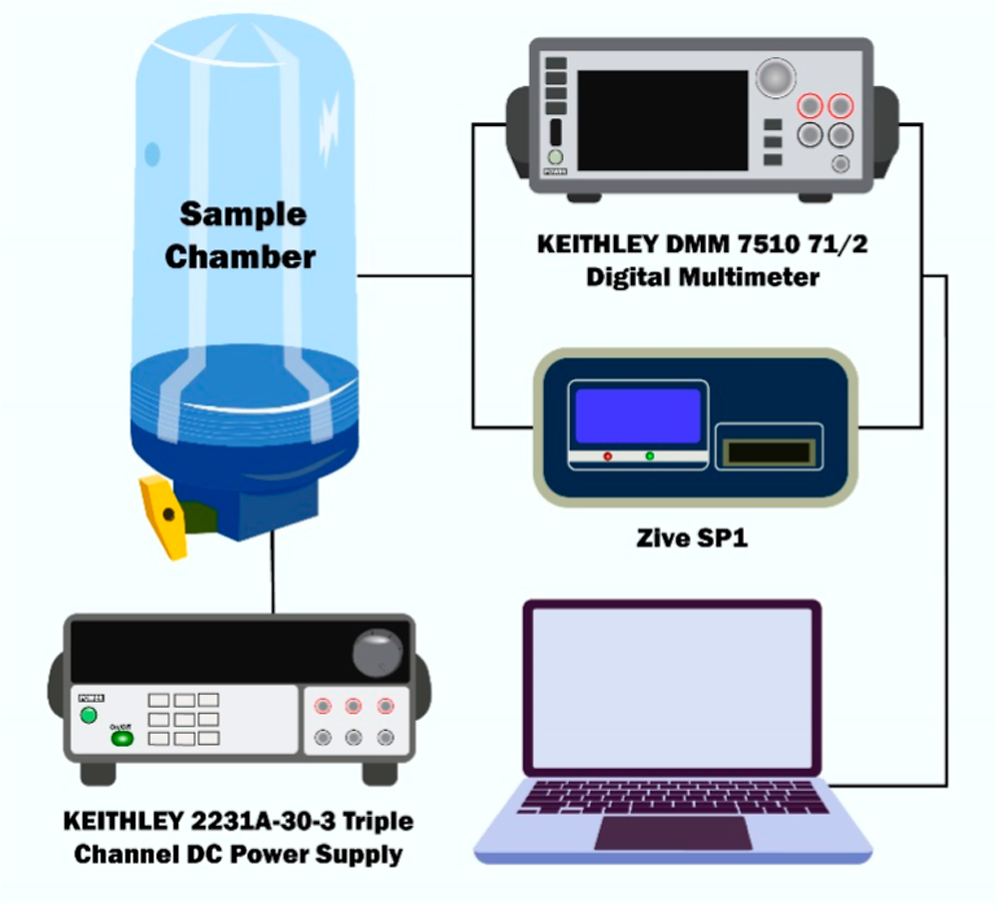
Illustration of the experimental setup.

The current–voltage, impedance, and response characteristics of the SnO_2_ ⟨Co⟩ sensor toward HPV were measured. The illustration of the experimental setup is presented in Figure 3.

In the hermetic experimental chamber, all necessary terminal contacts were prepositioned and ready for connection to the sensor pins. The sensor was placed inside the chamber on a special platform, which was connected to two recording devices. Real-time measurements of the electrical resistance of the sensor were carried out using a KEITHLEY DMM7510 7 1/2 high accuracy digital multimeter, while the monitoring data were recorded using KickStart 2 software. The current–voltage and impedance characteristics were measured by the Potentiostat/Galvanostat/EIS Wonatech Zive SP1 where current–voltage was studied over a voltage range of −2 to +2 V at a scan rate of 50 mV/s. The impedance measurements were done in a frequency range of 10^–1^–10^6^ Hz (with a sinusoidal signal of different amplitudes, under 1 V bias voltage). The results of the experiments mentioned above were recorded by the Smart Manager 6 software of the Zive SP1 device.

The measurements were carried out in the temperature range from room temperature to 200 °C. To heat the sensor, the appropriate voltage from the power supply (KEITHLEY 2231A-30-3) was applied to the heater. Before each measurement, the sample was heat stabilized keeping 45 min at the working temperature. The temperature deviation during the measurement processes did not exceed ±3 °C.

All measurements were carried out in the air and in the presence of different concentrations of HPV in the chamber. To create the appropriate gas concentration, after thermal stabilization of the sensor, a specified amount of aqueous H_2_O_2_ solution (corresponding to the required HPV concentration) was injected into the experimental chamber. This solution evaporated on the special platform (heated to ∼100 °C) inside the chamber to convert the H_2_O_2_ to its gaseous form. The gas-sensitive characteristics of the sensor were investigated toward 8–100 ppm of HPV concentrations.

The response of the sensor to HPV was investigated by three different measurements. First, the real-time monitoring of the sensor resistance was carried out, where the response was determined as the ratio of the electrical resistance in the presence of HPV and in the air, respectively. In the second case (current–voltage measurements), the sensor response was calculated as the ratio of the currents flowing through the sensor structure (for 1 V applied voltage) in air and in the HPV environment, respectively. Finally, the frequency characteristics of the impedance were used to calculate the response, which was determined as the ratio of the real components of the complex impedance obtained as a result of the measurements in the HPV environment and in the air.

## Results and Discussion

**Figure 4:**
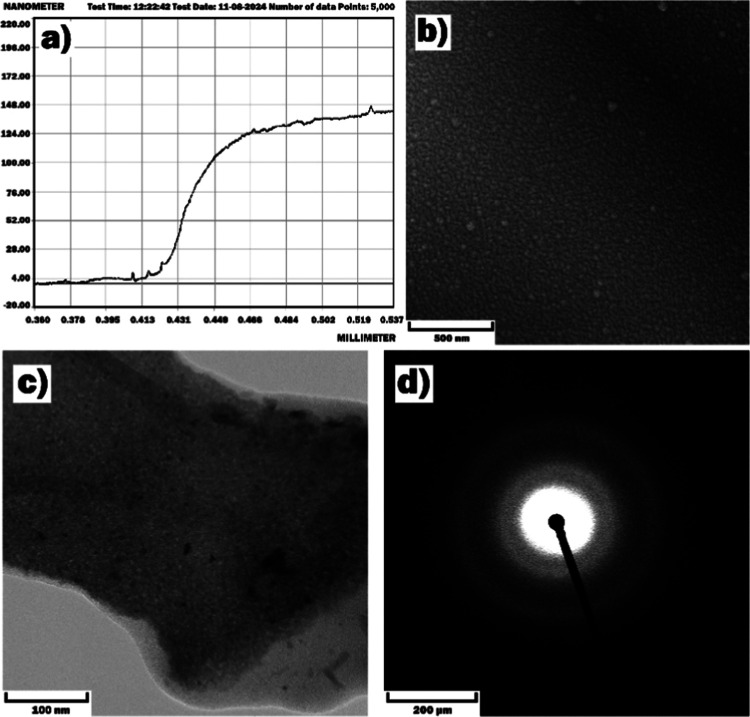
(a) Thickness measurement results, (b) SEM and (c) TEM images, and (d) SAED pattern of the SnO_2_ ⟨Co⟩ film.

The thickness of the SnO_2_ ⟨Co⟩ film deposited on the multisensor platform was measured revealing a thickness of approximately 130 nm (Figure 4a). The SEM, transmission electron microscopy (TEM), and selected area electron diffraction (SAED) analysis of the film showed that the grains with an average size of ∼20–25 nm (Figure 4b,d) were uniformly distributed, indicating its high compactness and near polycrystalline structure (Figure 4d).^52^

The gas-sensing studies of the SnO_2_ ⟨Co⟩ sensor were carried out using both the DC and AC conditions. In the first case, the electrical resistance and current–voltage characteristics of the sensor were measured, while in the second case, the frequency dependence of the complex impedance of the sensor was examined.

**Figure 5:**
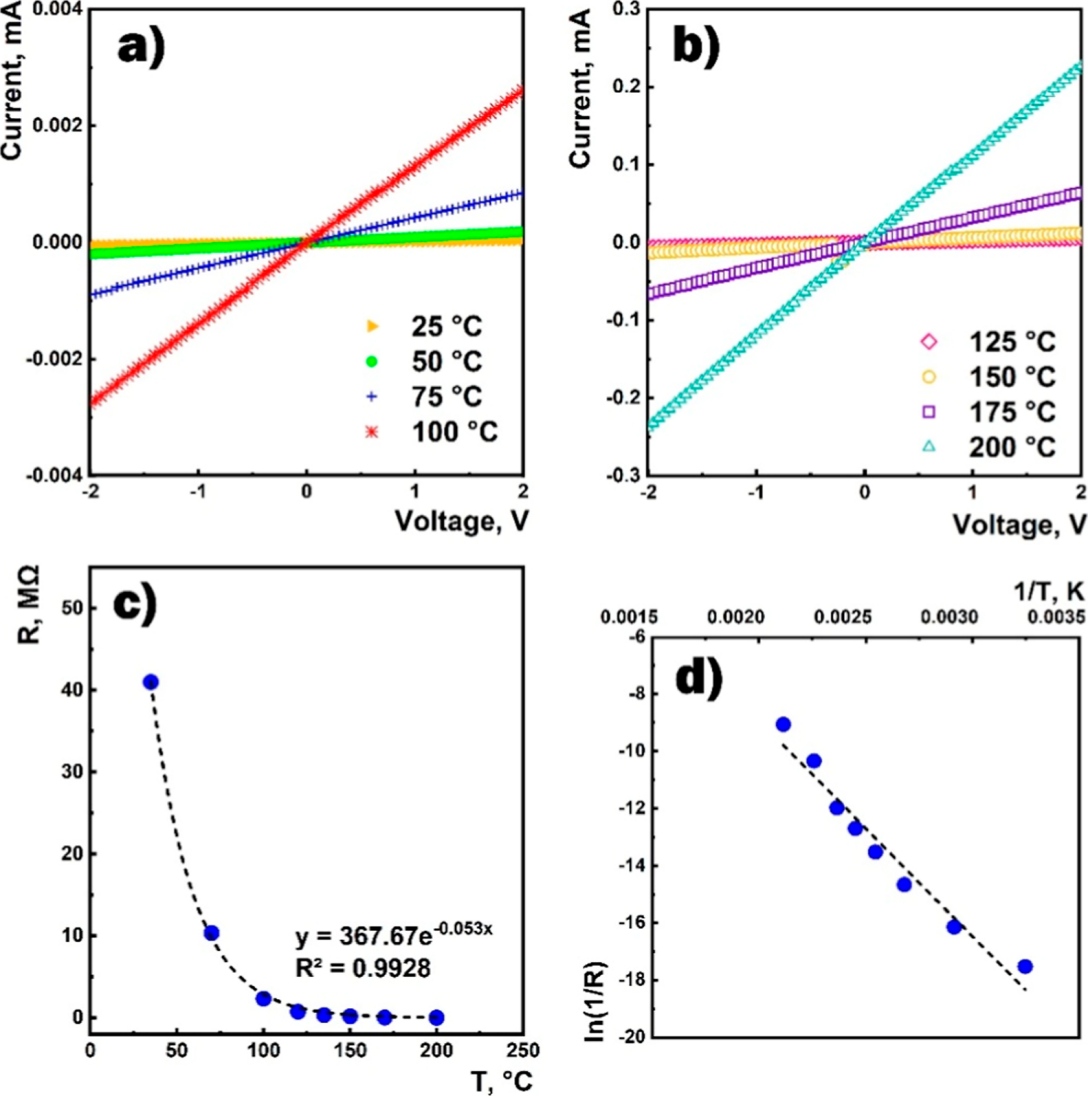
(a,b) Current–voltage characteristics of the sensor in the air at different temperatures, (c) dependence of the sensor resistance on the temperature, and (d) activation energy of the conductivity.

The current–voltage characteristics of the SnO_2_ ⟨Co⟩ sensor measured in the air at different temperatures are shown in Figure 5a,b. Using the obtained linear current–voltage characteristics, the resistance of the sensor (Figure 5c) and the activation energy of the conductivity (Figure 5d) were calculated. The sensor resistance was exponentially decreased with the increasing of the working temperature exhibiting about a 40 MΩ value at room temperature.

It is well-known that during high-temperature synthesis in polycrystalline SnO_2_, oxygen vacancies are formed, which act as shallow donor centers that are almost completely ionized at room temperature.^53,54^ Moreover, doping with the transition metal (cobalt) can lead to the formation of both shallow and deeper donor levels inside the band gap of SnO_2_, which are partially ionized at room temperature. As the charge carriers move through the film, they have to overcome two regions with different dielectric properties: the bulk and intergranular boundaries of the grains. The high resistance of the SnO_2_ ⟨Co⟩ film in the air at room temperature was attributed to the availability of the height of the potential barriers at the grain boundaries. The increase in the number of conduction electrons due to the rise in the operating temperature is related to the ionization of the neutral donors and the increase in the number of electrons with sufficient energy to overcome the intergrain energy barrier. The charge transfer process across the grain boundary is carried out through the hopping mechanism (transport). The activation energy of the conductivity calculated from the plot (Figure 5d) was approximately ∼0.65 eV.^54,55^ Besides, it is imperative to consider the impact of adsorption effects on the surface of the metal oxide. The adsorption of oxygen molecules on the surface can result in the capture of electrons from the SnO_2_ lattice, thereby increasing the resistance of the thin film. These processes exhibit high activity and can exert a substantial influence at temperatures above 150 °C.^56^

DC measurements provide information about the overall behavior of the sensor, assessing parameters, such as sensitivity, operation speed, and selectivity. However, to gain a deeper understanding of the gas-sensing mechanism, dynamic AC measurements are required. These measurements can also help to identify the factors and processes responsible for gas-sensitive properties and provide a quantitative assessment of the contributions bulk, surface, grain boundaries of the film, and electrode contacts and substrate of the sensor. To obtain a complete result, such measurements should be performed over a wide range of frequencies, temperatures, and environmental conditions. The analysis of the resulting impedance characteristics (AC measurement) involves the selection or construction of an equivalent electrical circuit that adequately reflects the electrical and chemical processes occurring in the system, the determination of the resistive and capacitive parameters of the equivalent circuit, and the calculation of the fitting frequency dependencies of the impedance.

**Figure 6:**
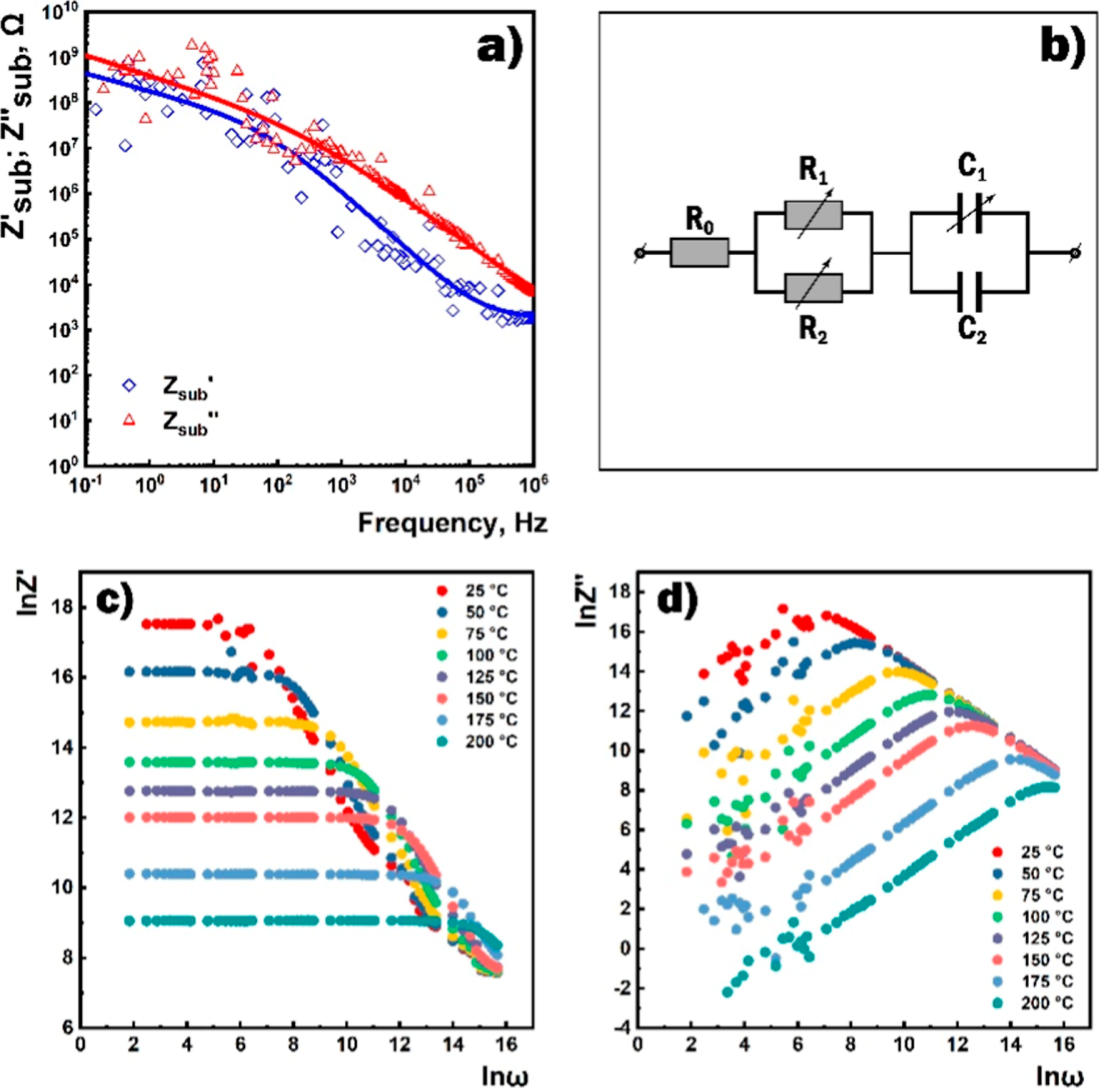
(a) Frequency dependencies of real (*Z*_sub_^′^) and imaginary (*Z*_sub_^″^) components of the substrate impedance (dotted values are experimental data and solid lines are theoretical calculations), (b) equivalent electrical circuit of the substrate, and frequency dependencies of the real (c) and imaginary (d) components of complex impedance at different temperatures.

Since we apply an alternating signal to the sensor influenced by a combination of many factors, the analysis of the impedance characteristics of any structure must take into account the contribution of all possible components of the system and the physicochemical processes occurring within it. As a typical structural component of the sensors, the dielectric substrate must be endowed with good dielectric properties, high adhesion, and high stability to elevated temperatures, aggressive gases, multiple thermal modulations, and surrounding electric and magnetic fields. Thus, such a substrate should contribute minimally to the impedance of the gas-sensing film. To fabricate the sensor, a metal oxide SnO_2_ ⟨Co⟩ film was sputtered on interdigitated platinum electrodes printed onto the substrate (Figure 2). At room temperature, the substrate exhibited a very high resistance (>1.2 GΩ). However, given the rather complex structure of the substrate, the impedance characteristics of the substrate without the gas-sensitive layer were preliminarily investigated to determine its possible contribution to the impedance of the fabricated sensor. The need for such studies and the consideration of the influence of the substrate on the impedance characteristics of sensors has been investigated by many researchers.^44^ The substrate impedance measurements were carried out in the temperature range of 25–240 °C, with external bias voltage ranges of 0–7 V and with an amplitude of the disturbing voltage of 10 to 300 mV. It was found that the course of the frequency dependences of the real (*Z*_sub_^′^) and imaginary (*Z*_sub_^″^) components of the complex impedance of the substrate remained unchanged under the influence of the above-mentioned parameters. Typical results of these measurements are shown in Figure 6a (the corresponding equivalent electrical circuit is shown in Figure 6b).

In the low-frequency range (lower than 100 Hz), the obtained characteristics, depending on the angular frequency ω, can be described by the following formulas derived from the curve depicted in Figure 6a for low-frequency dependencies of the real and imaginary components of the substrate impedance
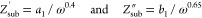
1where constants *a*_1_ and *b*_1_ are 4 × 10^8^ and 9 × 10^8^, respectively. In the low-frequency range, the main components contributing to the substrate impedance are the series-connected variable resistance *R*_1_ = *a*_1_/ω^0.4^ and capacitance *C*_1_ = 1/(*b*_1_ ω^0.35^). Since in this frequency range, the values of the real and imaginary components of the substrate impedance are in the same order (10^8^ Ω), it can be concluded that in the low-frequency range, the substrate does not make a significant contribution to the complex impedance of the sensor structure.

In the high-frequency range (>100 Hz), the impedance characteristics of the substrate are described by the following formulas derived from the curve depicted in Figure 6a for high-frequency dependencies of the real and imaginary components of the substrate impedance
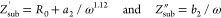
2where *R*_0_ ∼ 1500 Ω, *a*_2_ ∼ 2 × 10^10^, and *b*_2_ ∼ 5 × 10^10^. The primary active elements in this frequency range are the series-connected variable resistance *R*_2_ = *a*_2_/ω^1.12^ and constant capacitance *C*_2_ = 1/*b*_2_ ≈ 2 × 10^–11^ F. The constant resistance *R*_0_ makes a noticeable contribution to the real component of the impedance in the frequency range above 10^5^ Hz. This is probably due to the influence of the cables, as well as inductive effects associated with the experimental setup, which usually lead to an increase in the resistance value.^57^ The influence of these factors was then eliminated by an appropriate calibration. In the high-frequency range, the imaginary component of the impedance significantly exceeded the real component in the absolute value, which was why the module of the complex impedance was almost identical with the imaginary component. Therefore, the multisensor platform makes a significant contribution to the complex impedance of the sensor at high frequencies, mainly in the form of “parasitic” capacitance (*C*_2_).

Measurement results of the impedance characteristics of the SnO_2_ ⟨Co⟩ film carried out in the air at different temperatures with dependencies of the real (*Z*′) and imaginary (*Z*″) components of the complex impedance on frequency are demonstrated in Figure 6c,d, respectively. These dependencies show the transformation of the impedance characteristics of the sensor with an increase in temperature. The real component values decrease, while the slope angle of the imaginary component in the high-frequency range remains unchanged. Besides, the relaxation frequency (ω_0_) corresponding to the point of maximum of the imaginary component increases indicating the switching of currents between elements.

**Figure 7:**
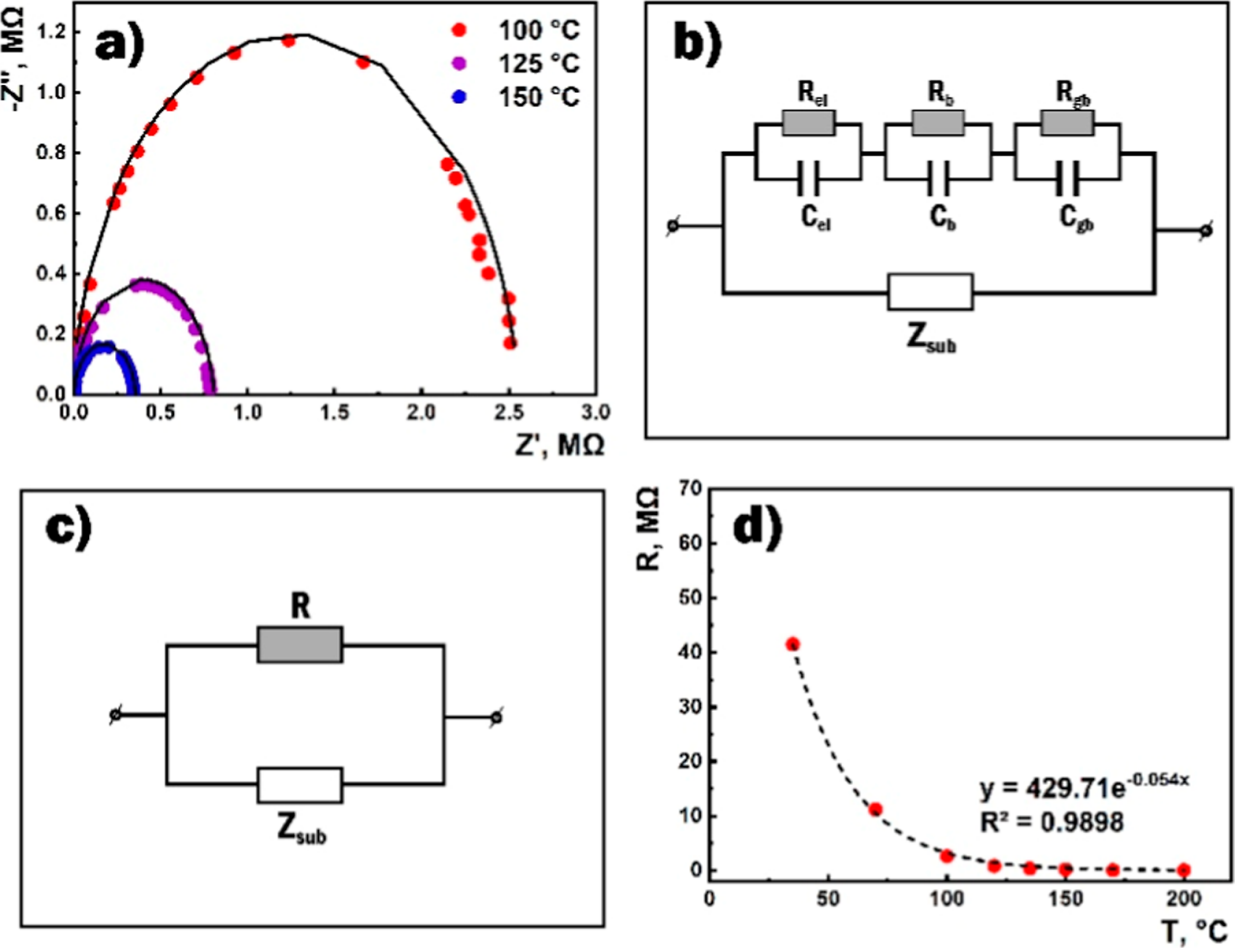
(a) Nyquist curves the SnO_2_ ⟨Co⟩ film in the frequency range above 100 Hz at different temperatures in the air (experimental data: dotted values, fitting data: solid lines), (b) equivalent electrical circuit of the sensor structure in the general case, (c) the equivalent electrical circuit for the SnO_2_ ⟨Co⟩ sensor at the high-frequency range, and (d) dependence of the sensor resistance (*R*) on the temperature.

The Nyquist plots obtained at different temperatures as a result of the impedance measurement have the form of semicircles in the high-frequency range (Figure 7a). This allowed us to assume that the structure in the high-frequency range can be modeled using parallel-connected R and C elements. The values of these elements were estimated as a behavior of the experimental frequency dependences of the real and imaginary components of the impedance. The value of *R* was the doubled value of the real component of the impedance at the frequency corresponding to the peak of the imaginary component. The value of *C* was estimated from the equation of ω_0_*RC* = 1. The values of *C* and *R* represented the high-frequency range corresponding the equivalent circuit presented in Figure 7 and the values of *C*_t_ and *R*_t_ assigned to the entire frequency range are summarized in Table 1. Here, the *C* capacitance is practically independent of the temperature, and its value is identical to the value of the substrate capacitance (*C*_2_). The increase in temperature only affected the *R* resistance (Figure 7d), whose behavior coincided with the dependence of the active resistance of the film on the temperature, obtained from measurements of the current–voltage characteristics of the sensor. Further investigation showed that *R* was mainly dependent on temperature, and its value was practically unaffected by variations in the external bias and the amplitude of the sinusoidal disturbance at the given temperature.

**Table 1:** Numeric Values of *C*, *R*, *R*_t_, and *C*_t_ at Different Temperatures

*T*, °C	*C* × 10^–11^, F	*R*, ohm	ν_0_, Hz	*C*_t_, F	*R*_t_, ohm
25	2.02	4.15 × 10^7^	1.9 × 10^2^	2.82 × 10^–10^	2.04 × 10^6^
50	2.22	1.12 × 10^7^	6.4 × 10^2^	8.37 × 10^–9^	3.62 × 10^5^
75	2.23	2.55 × 10^6^	2.8 × 10^3^	8.96 × 10^–9^	1.92 × 10^4^
100	1.98	8.03 × 10^5^	1 × 10^4^	6.02 × 10^–8^	2.09 × 10^4^
125	2.37	3.54 × 10^5^	1.9 × 10^4^	3.8 × 10^–4^	3.49 × 10^5^
150	2.14	1.62 × 10^5^	4.6 × 10^4^	9.5 × 10^–4^	1.63 × 10^5^
175	2.78	3.02 × 10^4^	1.9 × 10^5^	5.8 × 10^–3^	2.97 × 10^4^
200	2.74	7.1 × 10^3^	8.2 × 10^5^	4.4 × 10^–2^	4.4 × 10^3^

The modeling of the equivalent electrical circuit of a sensor structure takes into account the contribution of each of the numerous factors that determine the system impedance. Thus, taking into account the contribution of the substrate, the equivalent electrical circuit of a sensor should consist of two parallel directions. One reflects the contribution of the gas-sensing layer and the other is due to the substrate (Figure 7b). The equivalent circuit that describes the behavior of the polycrystalline film contains three series-connected subcircuits. Each of them consists of a parallel-connected resistor and a capacitor characterized by the time constant.^44,56,57^ This is because charge carriers transferring through the polycrystalline film have to overcome the series impedances of three different regions: the bulk of grain, the grain boundary, and the contact electrode, which have various dielectric properties. Therefore, the main contributors to the impedance are intragrain (bulk), intergrain (grain boundary), and electrode (film–electrode contact) components, each of which is described by an *RC* element and by three semicircles corresponding to the Nyquist curve. The low-frequency contribution of the *R*_el_*C*_el_ element associated with electrode processes can be ignored, as the linear current–voltage characteristics over the entire temperature range indicate the Ohmic behavior of the platinum contacts, which may be a consequence of the high concentration of charge carrier in the SnO_2_ ⟨Co⟩ film. In practice, the bulk capacitance is significantly less than the capacitance of various junctions. Therefore, the contribution of processes in the grain bulk is usually characterized by the active resistance (*R*_b_), the temperature dependence of which is mainly related to thermal ionization of the carriers. The resistance *R*_gb_ and capacitance *C*_gb_ of the grain boundary should be greater than those of the grain bulk, and both should decrease with increase in temperature due to the activation of the hopping mechanism. The absence of the second semicircle on the experimental impedance curves indicates a superposition of the influences of the processes occurring in the grain bulk and at the grain boundaries since the response obtained from different parts of the sensitive layer (from different RC circuits) can be separated from each other if the corresponding relaxation times differ by more than an order of magnitude. Thus, according to the experimental data obtained in the frequency range above 100 Hz, the sensor structure can be represented by the equivalent electrical circuit shown in Figure 7c. In this case, the resistance is *R* ≈ *R*_b_ + *R*_gb_. The dominant substrate capacitance does not allow for the detection of the grain boundary *C*_gb_ capacitance. Fitting impedance dependencies in the high-frequency range were calculated according to the formulas
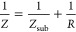
3
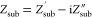
4

The values of *R* (Table 1) and the measured values of the real (*Z*_sub_^′^) and imaginary (*Z*_sub_^″^) components of the substrate impedance (eq 2) were used in the calculations. It was shown that in the case of SnO_2_ nanoparticles with sizes smaller than 25 nm, they are almost completely depleted. Consequently, the transfer processes of the carriers should be mainly determined by the intercrystallite boundaries.^55^ Since the average grain size in the SnO_2_ ⟨Co⟩ film is ∼20 nm, it can be assumed that the resistance *R* is mainly defined by the grain boundary resistance.

**Figure 8:**
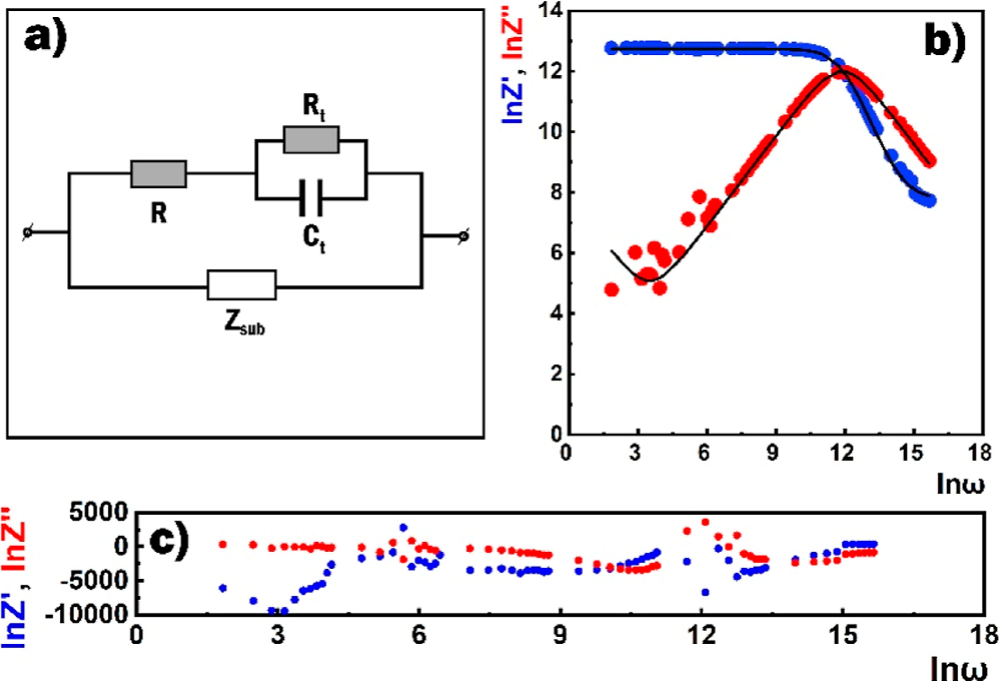
(a) Equivalent electrical circuit of the SnO_2_ ⟨Co⟩ sensor for the whole frequency range, (b) frequency dependencies of real and imaginary components of impedance at 125 °C in the air (the dotted values represent the experimental data, and the solid lines are fitting curves), and (c) distribution of the fitting absolute error.

ZMAN 2.3 software was used to analyze the measured impedance characteristics of the SnO_2_ ⟨Co⟩ structure in the frequency range below 100 Hz. It was found that in the low-frequency range, the contribution to the total impedance of the investigated sensor is given by the *R*_t_*C*_t_ element, the parameters of which are given in Table 1. It is assumed that this process may be related to the formation of the deeper energetic states inside the band gap of SnO_2_ due to the cobalt doping. These energetic states may act as electron trapping centers, which can be present in both the bulk and the boundary region of the grain. Besides, existing defects on the grain surface can also act as traps, capturing and rereleasing some electrons. This process contributes to the impedance at low frequencies and leads to a large increase in capacitance.^44,57^ If the frequency becomes too high (>50 Hz), these traps are unable to keep up with the high frequency and their charge–discharge processes cease to play a dominant role in charge transfer processes. Thus, the resulting fitting impedance curves of the SnO_2_ ⟨Co⟩ sensor over the entire frequency range of measurements were calculated for the equivalent electrical circuit shown in Figure 8a, according to the following formulas
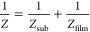
5
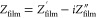
6
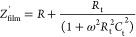
7
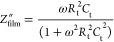
8

The calculations were made using the data in Table 1 and taking into account the substrate impedance values (*Z*_sub_^′^ and *Z*_sub_^″^) obtained from experimental measurements. Figure 8b shows the frequency (experimentally measured) dependence of the real and imaginary components and the calculated fitting curves over the entire frequency range. A sufficiently good agreement of the experimental data with the theoretically calculated dependences is evidenced by the distribution of the absolute error (Figure 8c), proving the reliability of the chosen equivalent electrical circuit to describe the processes occurring in the fabricated SnO_2_ ⟨Co⟩ sensor.

**Figure 9:**
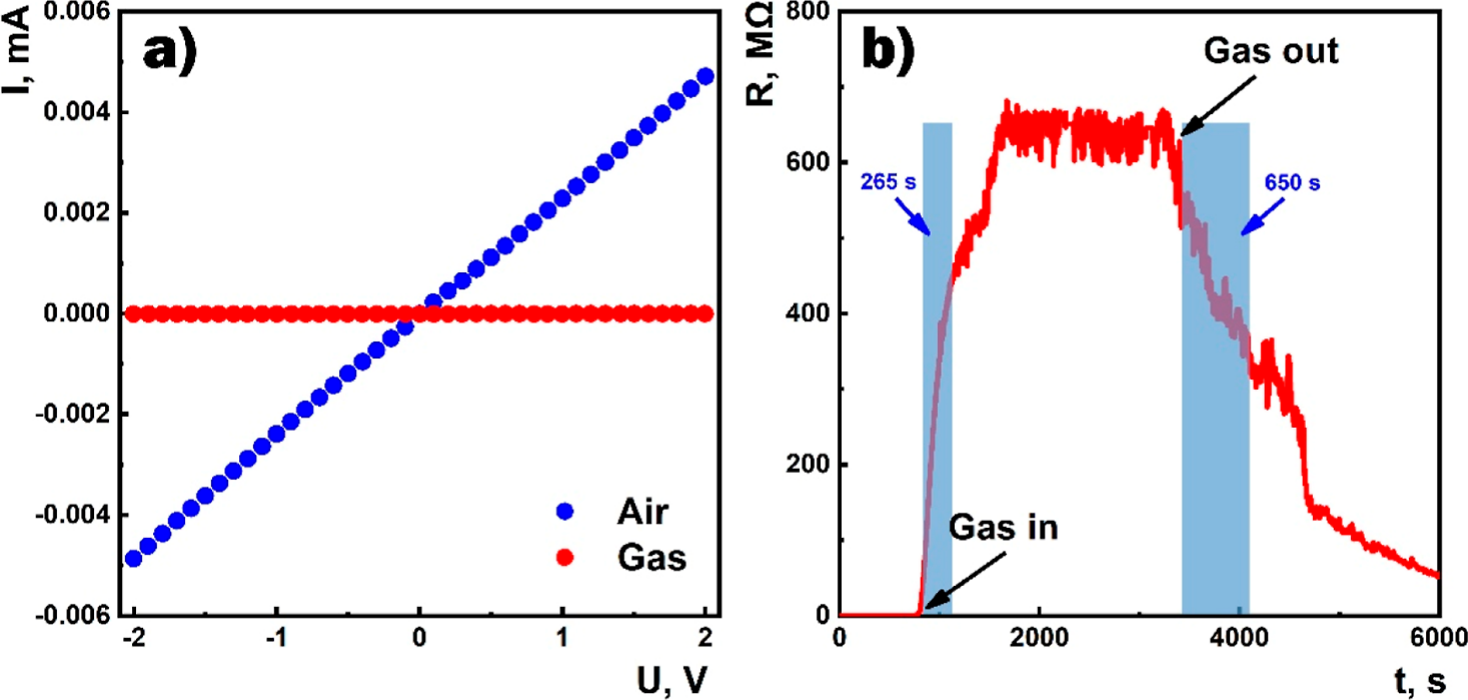
(a) Current–voltage characteristics and (b) real-time resistance change of the SnO_2_ ⟨Co⟩ sensor in the air and the presence of 100 ppm of HPV at 125 ^°^C.

The sensor response to HPV was investigated by measuring its current–voltage characteristics after exposure to the target gas as well as by direct resistance monitoring (Figure 9). Under atmospheric conditions, oxygen species (O_2_, O^–^, and O_2_^–^) are adsorbed onto the surface of the semiconductor film from the environment, taking free electrons from it and saturating the surface with a depleted layer. Upon the influence of HPV, there was a significant increase in the active resistance of the film, which led to a sharp decrease in the current. The chemiresistive sensing mechanism of H_2_O_2_ on the metal oxide’s surface is well-known.^37,38,58^ Here, H_2_O_2_ molecules tend to split into water and oxygen molecules on the surface of the SnO_2_ ⟨Co⟩ film capturing electrons from the semiconductor lattice and transforming into oxygen species in the ionic state (eqs 9–11).^59^
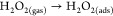
9
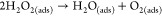
10
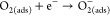
11

**Figure 10:**
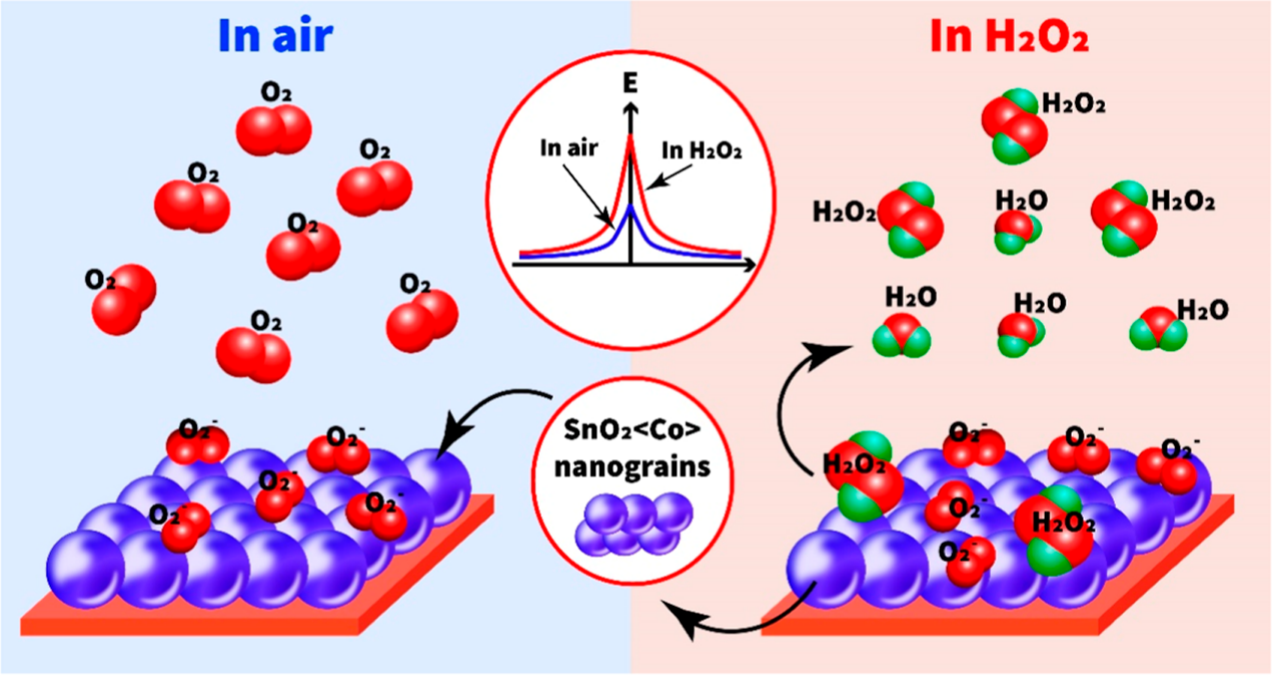
Schematic block diagram of the HPV-sensing mechanism.

This electron exchange results in an increase in the sensor resistance (Figure 10). The sensor responses (currents ratio in the air and HPV, respectively) to 100 ppm of HPV sensor at applied 1 V voltage were 41, 746, and 841 at the operating temperatures of 75 °C, 100 °C, and 125 °C, respectively.

Real-time monitoring of the sensor resistance allowed us to establish a reasonably fast HPV-sensing behavior. At room temperature, when the sensor resistance increased only three times, it took about 30 s. This response time decreased as the operating temperature increased reaching less than 10 s at 125 °C. Besides, the response and recovery times along the baseline trace of the response curve were assigned 265 and 650 s, respectively (Figure 9b). The recovery time was quite long, which can be attributed to the slow desorption rate of the HPV molecules at relatively low temperatures. The responses of the SnO_2_ ⟨Co⟩ sensor calculated from the change in active resistance were about 64, 1175, and 1321 at temperatures of 75 °C, 100 °C, and 125 °C, respectively. The low response values obtained in current–voltage measurements are due to this circumstance. Rapid pulsed heating in ranges higher than the main operating temperature can lead to the acceleration of desorption processes.

**Figure 11:**
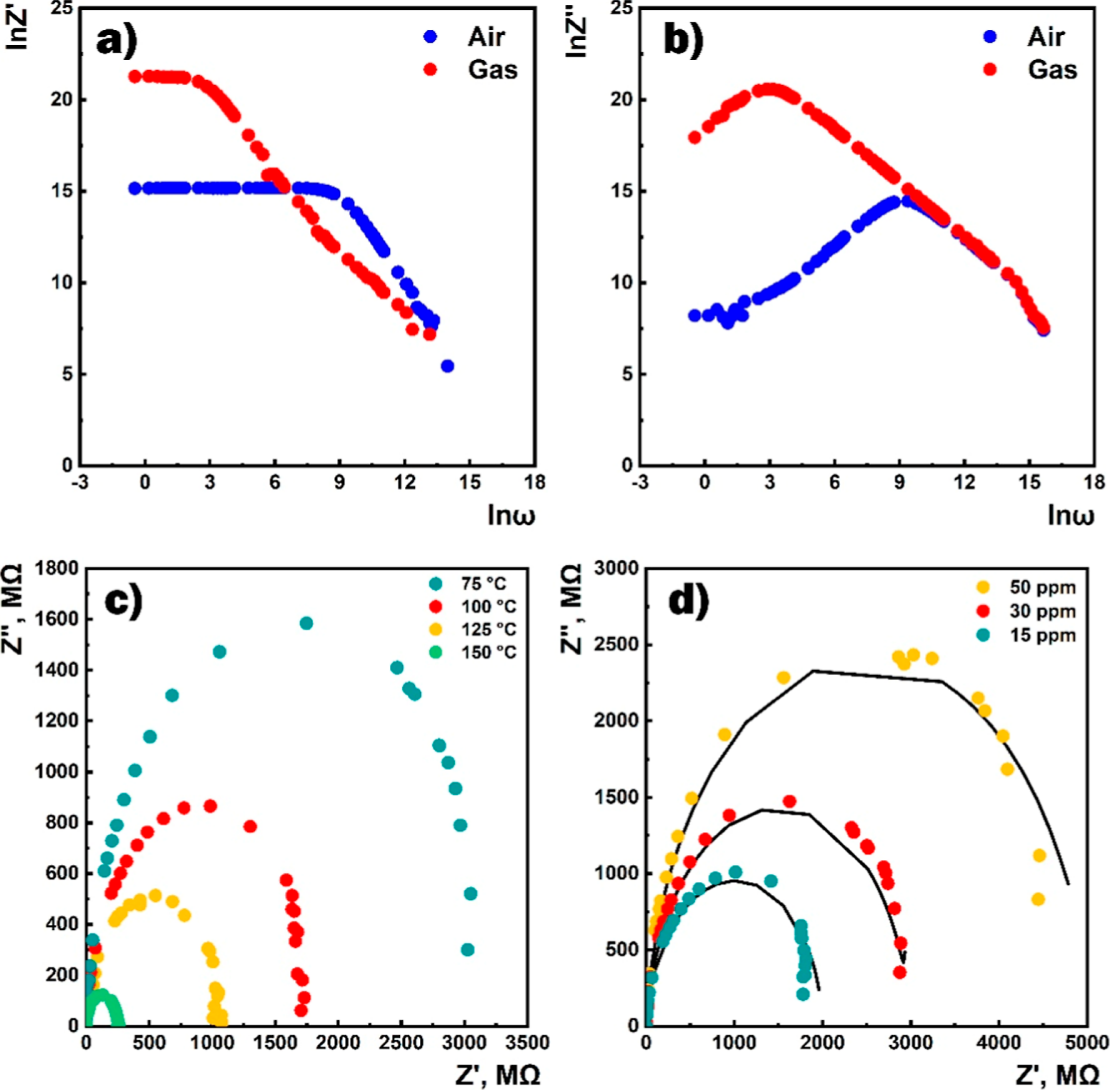
Frequency dependencies of (a) real and (b) imaginary components of impedance in the air and the presence of 100 ppm of HPV at 100 °C, (c) Nyquist curves of the sensor in the presence of 100 ppm of HPV at different temperatures, and (d) Nyquist curves of the sensor for different concentrations of HPV at 50 °C (dotted values: experimental data; solid lines: fitting curves).

Figure 11a,b shows a typical deviation of the frequency dependence of the real and imaginary components of the complex impedance of the sensor under the influence of 100 ppm of HPV. Upon exposure to the target gas, an increase in the real component in the low-frequency range and consequently a shift of the relaxation frequency toward low frequencies were observed. The increase in the operating temperature of the sensor in air was accompanied by the deviation of the Nyquist semicircle toward the low resistance region (Figure 11c). The effect of different concentrations of HPV on the impedance characteristics showed an increase in the Nyquist semicircle radius with increasing target gas concentration (Figure 11d). The equivalent electrical circuit shown in Figure 8a was used to model the sensor structure. The fitting curves, which were calculated using the parameter values at 50 °C (Table 2) obtained by the ZMAN 2.3 software, fit the experimental characteristics quite well.

**Table 2:** Parameter Values of the Equivalent Circuit Elements at 50 °C

HPV concentration, ppm	in air	in HPV	in air	in HPV	in air	in HPV
	*R*, ohm	*R*, ohm	*C*_t_, F	*C*_t_, F	*R*_t_, ohm	*R*_t_, ohm
8	2.64 × 10^7^	5.05 × 10^8^	1.78 × 10^–6^	5.45 × 10^–8^	1.81 × 10^12^	9.23 × 10^12^
16	1.52 × 10^7^	1.87 × 10^9^	4.73 × 10^–6^	9.36 × 10^–9^	1.48 × 10^11^	4.89 × 10^11^
33	2.7 × 10^7^	3.46 × 10^9^	4.31 × 10^–6^	4.26 × 10^–9^	4.32 × 10^11^	1.98 × 10^11^
50	2.63 × 10^7^	4.75 × 10^9^	5.20 × 10^–6^	2.54 × 10^–9^	6.32 × 10^11^	2.71 × 10^11^
100	7.38 × 10^6^	4.30 × 10^9^	6.21 × 10^–6^	1.75 × 10^–9^	1.27 × 10^11^	3.38 × 10^11^

The influence of the target gas led to a significant increase in the value of parameter *R* (Table 2), which mainly characterizes the resistance of the grain boundaries. The exposure of the target gas to the surface of the semiconductor film, accompanied by the exchange of electrons between adsorbed HPV molecules and the SnO_2_ ⟨Co⟩ lattice, leads to an even larger depletion region of the grain boundaries. As a result of these processes, the change in the film resistance was recorded revealing the change in the value of the *R* parameter. It should also be noted that the influence of HPV affected the parameters of the *R*_t_*C*_t_ element, which also led to an increase in the impedance, but its quantitative contribution to the response was significantly smaller.

**Figure 12:**
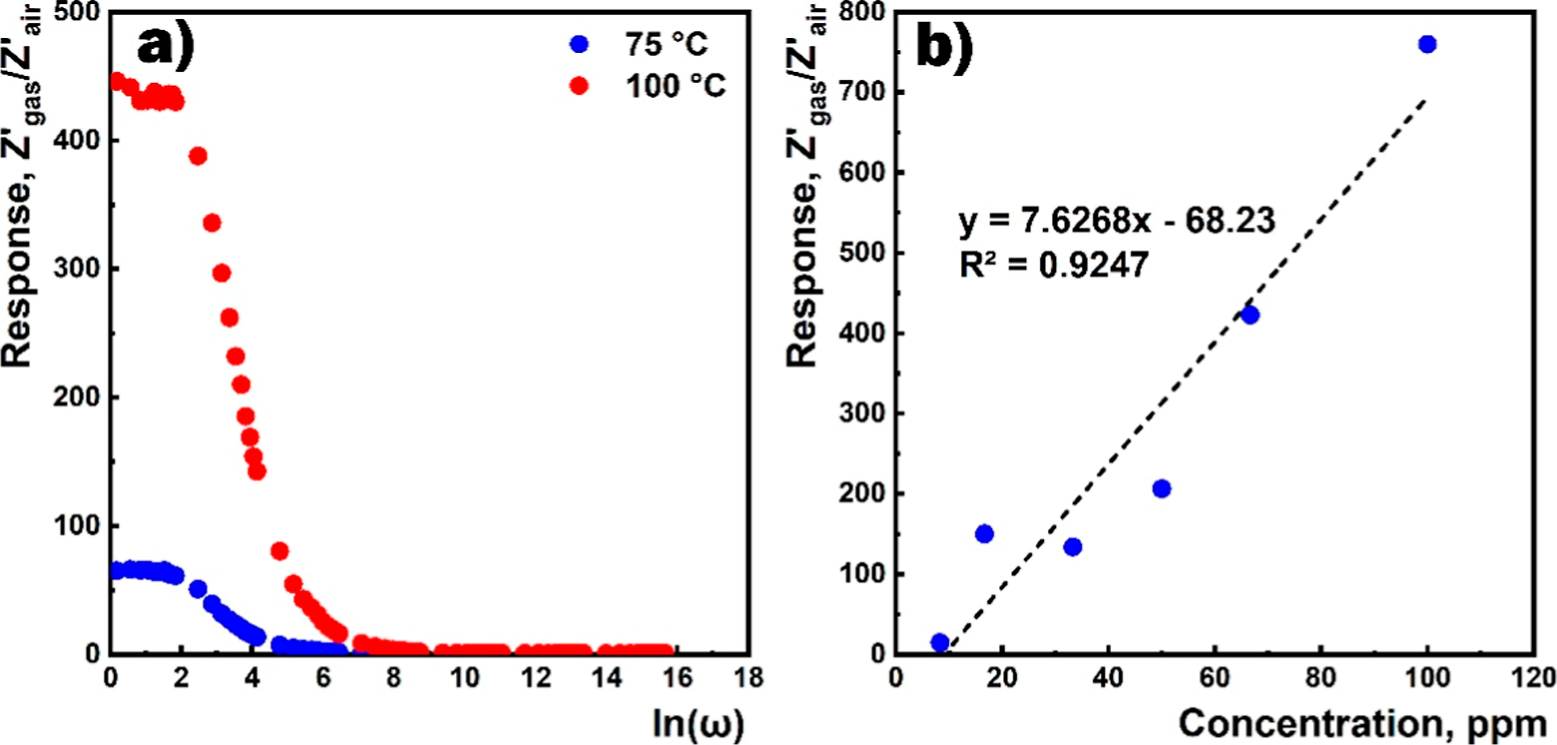
(a) Response vs frequency at different temperatures and (b) response vs HPV concentration at 50 °C.

The dependence of the sensor response on the frequency at different temperatures as well as on the HPV concentration at 50 °C is shown in Figure 12. As evident, the response did not change in the low-frequency range (less than 10 Hz). Besides, the dependence of the response on the HPV concentration at 50 °C was extrapolated by the linear function.

A brief literature survey was conducted comparing the characteristics of our and existing HPV sensors. As can be seen from Table 3, our sensor is actually comparable in its parameters, where a higher response value is particularly pronounced.

**Table 3:** Comparison Table between Our and the Previously Fabricated HPV Sensors Reported in the Literature

sensing materials	operating temperature [°C]	HPV concentration [ppm]	response	references
ZnO⟨La⟩	175	105	114	(47)
ZnO/La	240	100	13.7	(58)
MoS_2_/reduced graphene oxide (RGO)	RT	50	12	(60)
silver/gold metallic nanoparticles	RT	100	50%	(61)
MnO_2_/polyimide	140	20	30%	(62)
Fe_2_O_3_/ZnO nanograins	150	100	1550	(59)
SnO_2_ ⟨Co⟩ nanograins	125	100	1321	this work
SnO_2_ ⟨Co⟩ nanograins	25	1	2.2	this work

## Conclusions

This study has demonstrated the synthesis and impedance investigation of cobalt-doped SnO_2_ thin film obtained by the high-frequency magnetron sputtering method for the detection of HPV. The film thickness was approximately 130 nm, and the average grain size was in the range of 20–25 nm. The SnO_2_ ⟨Co⟩ sensor was investigated by complementary DC and AC measurements. The sensor responses to 100 ppm of HPV calculated from the change in active resistance were 64, 1175, and 1321 at the operating temperatures of 75 °C, 100 °C, and 125 °C, respectively. An equivalent electrical circuit of the investigated sensor was proposed based on the analysis of the frequency characteristics of the complex impedance. The sensor substrate made a significant contribution to the complex impedance of the sensor at high frequencies (>100 Hz), mainly in the form of “parasitic” capacitance, while in the low-frequency range (<100 Hz), the effect of the substrate was absent. It was found that the availability of the parallel capacitance of the substrate complicated to reveal the capacitance of the sensitive film. In the SnO_2_ ⟨Co⟩ film, the modulation of the space charge region at the grain boundaries was mainly responsible for the sensitivity to the target gas. The SnO_2_ ⟨Co⟩ nanostructured sensor can be considered a promising composite for the detection of low concentrations of HPV at rather low operating temperatures.
